# Anti-TIF1γ-Dermatomyositis and Sjögren’s Syndrome As the Inaugural Presentation for Rectal Cancer

**DOI:** 10.7759/cureus.55978

**Published:** 2024-03-11

**Authors:** Miguel Oliveira Santos, Inês Santos, Guilherme Sacramento, Rita Oliveira, Andrea Castanheira

**Affiliations:** 1 Oncology, Instituto Português de Oncologia de Lisboa Francisco Gentil, Lisbon, PRT; 2 Internal Medicine, Centro Hospitalar Lisboa Ocidental, Lisbon, PRT; 3 Pathology, Centro Hospitalar Lisboa Ocidental, Lisbon, PRT

**Keywords:** multidisciplinary team, dermatomyositis, anti-tif1-γ antibodies, paraneoplastic syndromes, rectal cancer, sjogren syndrome

## Abstract

Dermatomyositis (DM) is an inflammatory myopathy often paraneoplastic in nature. Patients have characteristic cutaneous findings and possible muscle involvement. In the latter, muscle enzymes are elevated, and the electromyogram shows varied changes. Muscle or skin biopsy and myositis-specific antibodies confirm the diagnosis. Here, we report the case of an 86-year-old woman with cutaneous lesions, proximal weakness, and sicca symptoms. Muscle enzymes and electromyogram were normal. Antinuclear antibodies were elevated, and anti-TIF1γ and anti-Ro52 antibodies were positive. Muscle biopsy was compatible with the diagnosis of DM, and salivary gland biopsy confirmed Sjögren's syndrome. Malignancy investigation identified a rectal cancer, which was resected. This case illustrates a rare form of cancer presentation - anti-TIF1γ DM with normal muscle enzymes and electromyogram and concomitant secondary Sjögren's syndrome. Malignancy screening and multidisciplinary management were crucial to a successful approach.

## Introduction

Cancer is responsible for a significant burden [1]. Early detection is important to improve prognosis. Besides typical symptoms linked to each tumor, it is important to identify other ways in which cancer can present, including immune-mediated processes prompted by cancer, namely, paraneoplastic syndromes [2,3].

Dermatomyositis (DM) presents with dermatologic alterations, and in 80% of the cases (classic DM), proximal and symmetric muscle weakness is present, whereas 20% have no muscle complaints (clinically amyopathic DM) [4,5]. Blood workup typically reveals elevated muscle enzymes, and electromyogram identifies small-amplitude, short-duration polyphasic motor units and fibrillation potentials [6,7]. Muscle biopsy abnormalities, including degeneration, regeneration, necrosis, phagocytosis, and interstitial mononuclear infiltrate, confirm the existence of inflammatory myopathy (IM). Typical myositis-associated and myositis-specific antibodies provide further evidence to distinguish between subtypes of IM [8]. In people over 40 years old, there is a great risk of malignancy (4.6- to 6-fold higher than the general population) [9,10]. Management of malignancy-associated DM is difficult to attain, with no guidelines defining the best timing for either cancer-directed surgery or glucocorticoid/immunosuppressive therapy [11,12].

Sjögren’s syndrome is a chronic autoimmune disease that affects predominantly women characterized by xerostomia and xerophthalmia (keratoconjunctivitis sicca)[4]. It can be primary, but most of the time, it is secondary, i.e., associated with other autoimmune diseases. Patients with autoimmune diseases suffer from Sjögren’s syndrome in 5-20% of cases [4]. The diagnosis is established by identifying glandular damage (sialometry, Schirmer's test, echography, or MRI) and autoimmunity (autoantibodies and salivary gland biopsy).

## Case presentation

An 86-year-old woman, with a known history of hypertension and subclinical hypothyroidism, presented with a 13-month history of progressive hand and facial pruriginous rash, alopecia, tiredness, and muscle weakness in her lower limbs. Xerostomia and oral aphtosis were also reported. She admitted having been exposed to dust in the last months but denied any other triggers. Her general practitioner prescribed a short course of low-dose oral and topical corticotherapy for the rash, with scarce improvement.

**Figure 1:**
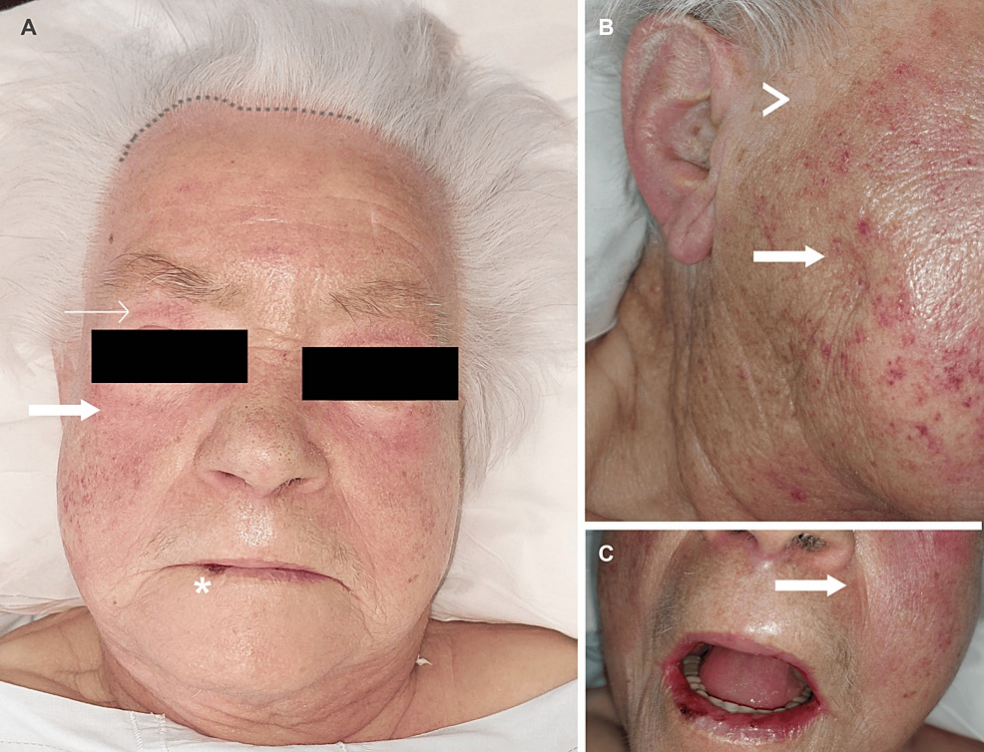
Dermatomyositis facial lesions. A: diffuse facial edema along with periorbital heliotrope rash (narrow arrows), malar rash without sparing de nasolabial folds (bold arrows), alopecia (irregular dotted line), and cheilitis (asterisk); B: rash in detail (bold arrows) and poikiloderma (arrowhead), C: detailed malar rash without sparing of the nasolabial folds (bold arrow)

**Figure 2:**
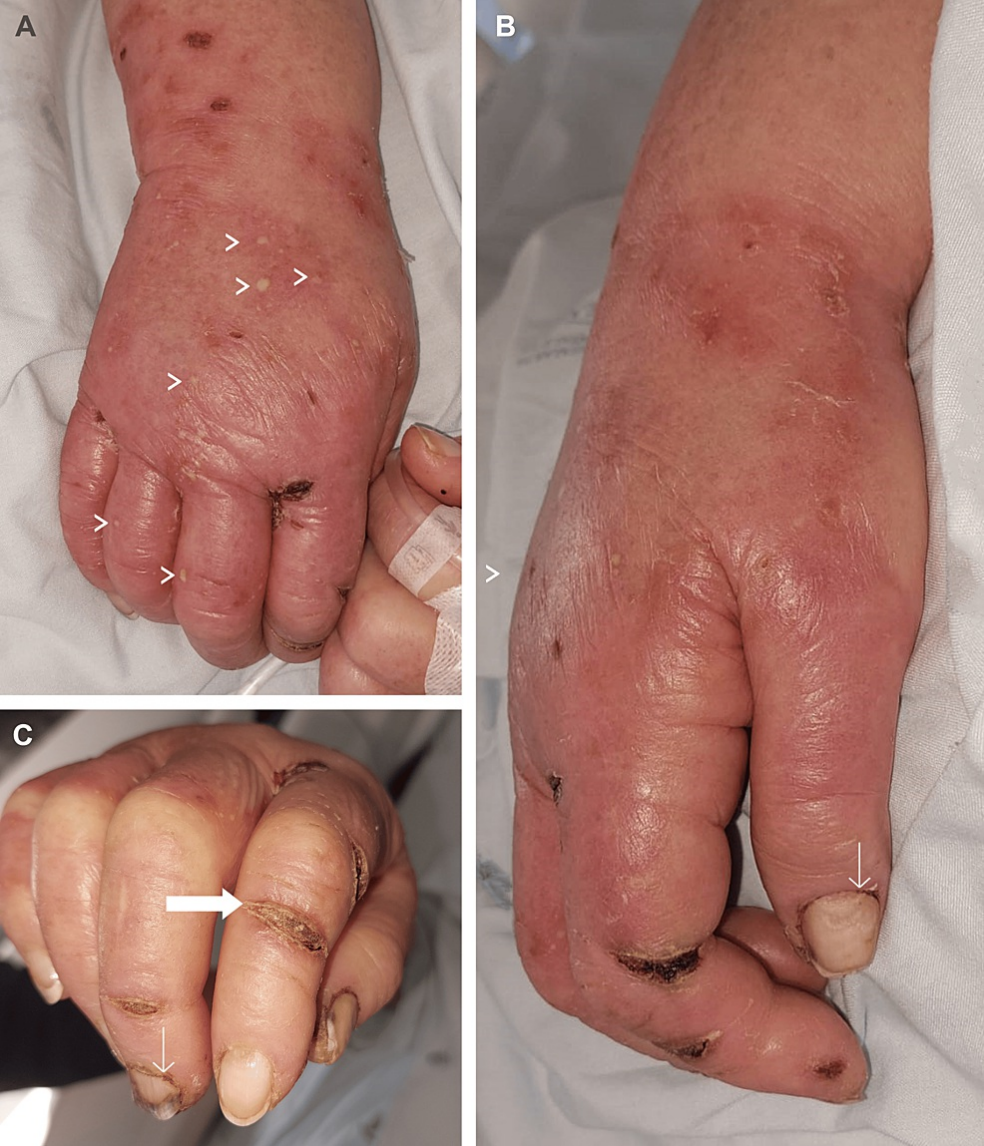
Dermatomyositis peripherical lesions. A: hand lesions showing diffuse rash and edema, pustules on the dorsal surface of the hands (arrowhead); B: detailed erythematous edema with fissuring of the finger along the radial aspect of the second and third fingers (mechanic hands) and dystrophic and ragged cuticles (Samitz' sign) (narrow arrow); C: erythematous fissures of mechanic hands (bold arrows) and subungual hemorrhages (narrow arrow)

On examination, she presented periorbital heliotrope and malar rash without nasolabial fold sparing, alopecia, scalp erythema with abnormal capillary implantation line (Figure 1A, 1B, 1C), and depapillated tongue. On the superior limbs, there was a bilateral edematous rash with thickening and ulcers in the hands, compatible with mechanic hands (Figure 2A, 2B, 2C). Cardiovascular, pulmonary, and abdominal examinations were normal. Neurologic examination revealed symmetrically diminished strength in proximal muscles (shoulder abductors and hip flexors).

Blood investigation demonstrated microcytic hypochromic anemia and subclinical hypothyroidism (Table 1). Muscle enzymes were within normal range, including creatinine kinase, aspartate aminotransferase, alanine aminotransferase, and lactate dehydrogenase. White blood count, C-reactive protein, electrolytes, and renal and hepatic function were normal.

**Table 1:** Blood investigation at admission. Apart from anemia and subclinical hypothyroidism, no other relevant changes were found in renal and hepatic function, muscle enzymes, ionogram, or C-reactive protein.

	Patient values at admission	Normal range
Hemoglobin (g/dL)	10.5	12.0-15.0
Leucocytes (cells/μL)	5,700	4,000-10,000
Platelets (cells/μL)	267,000	150,000-400,000
Aspartate aminotransferase (U/L)	34	<32
Alanine aminotransferase (U/L)	22	<33
Creatinine kinase (U/L)	106	<170
Lactate dehydrogenase (U/L)	183	135-225
Creatinine (mg/dL)	0.8	0.50-0.90
Urea (mg/dL)	71	17-49
Sodium (mmol/L)	136	136-145
Potassium (mmol/L)	4.61	3,50-5,10
Chlorine (mmol/L)	99	98-107
Bilirrubin (mg/dL)	0.28	<0.9
Albumine (g/dL)	4	3.5-5.2
Thyroid-stimulating hormone (μUI/mL)	8.72	0.27-4.20
Free thyroxine (T4) (pmol/L)	13.1	12.0-22.0
C-reactive protein (mg/dL)	0.74	<0.50

The patient was admitted to the inpatient ward for further investigation and treatment.

Clinical investigation

DM Investigation

Anti-nuclear antibodies (ANA) titers were high (>1:1,280), with a mottled nuclear pattern. High positivity was found for anti-TIF1γ - a malignancy-related myositis-specific antibody. Myositis-associated antibodies revealed positivity for anti-Ro52 (anti-SSA).

Electromyography showed mild S1 radicular impairment, without myopathic or polyneuropathic involvement.

**Figure 3:**
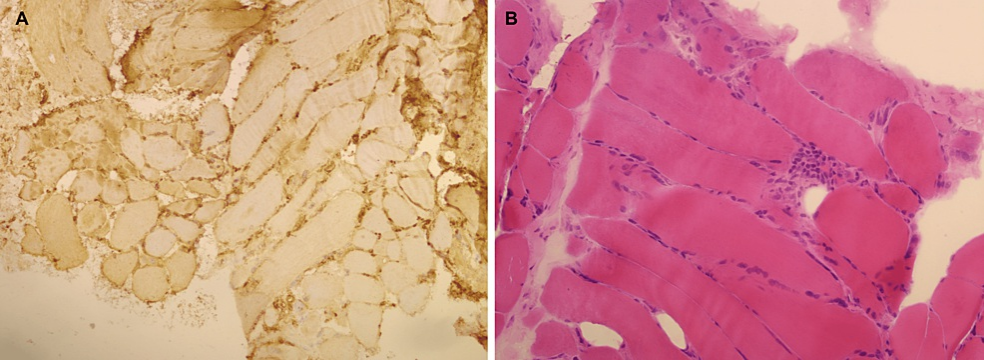
Muscle biopsy of the left quadriceps. A: immunohistochemical study showing global staining of muscle fibers for MHC-I. B: hematoxylin-eosin staining showing a necrotic muscle fiber, undergoing myophagocytosis, and variation of the muscle fiber size.

The muscle biopsy of the left quadriceps was performed, showing findings suggestive of inflammatory myopathy, namely, MHC-1 diffuse staining of muscle fibers (Figure 3A), variation of muscle fibril size, and necrosis due to the myophagocytosis process (Figure 3B). However, no pathognomonic findings of DM were found.

As our patient met the criteria for inflammatory myositis (i.e., had heliotrope rash; symmetric progressive weakness of the lower and proximal extremities, and myositis-specific antibodies and compatible biopsy), we assumed the diagnosis of DM. The diagnosis was confirmed after consulting with Rheumatology and Dermatology.

Sjögren’s Syndrome Investigation

As described, the patient also had sicca symptoms - xerostomia and xerophthalmia - for the previous year. When further questioned, she revealed to feel a burning mouth sensation and frequent oral aphthous ulcers. The examination also revealed a depapillated dry tongue and cheilitis (Figure 1).

**Figure 4:**
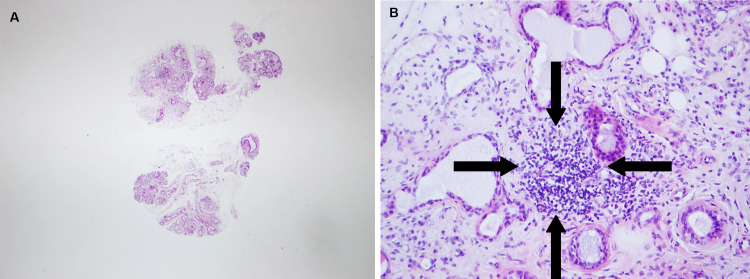
Salivary gland sample showing lymphocytic infiltrations (aggregate >50 lymphocytes/4 mm2) and interstitial fibrosis (hematoxylin-eosin staining). A: small magnification, B: high magnification. Black arrows show lymphocytic infiltration.

As referred before, she was positive (highly positive) for anti-Ro52 (anti-SSA). Minor salivary gland biopsy showed a focus on lymphocytic infiltration and interstitial fibrosis - Chisholm-Mason grade 3/4 (Figure 4A, 4B). A secondary Sjögren’s syndrome diagnosis was made [13].

Malignancy Investigation

According to guidelines, cancer screening must be carried out if there are risk factors associated. Clinical risk factors include onset in older ages or in male individuals, if there is a rapid disease onset, if dysphagia or cutaneous necrosis is present, or if the disease is refractory to immunosuppressive therapy [11]. Analytic criteria include positivity for anti-TIF1γ or anti-NXP2 antibodies or negativity for any known myositis-specific antibodies. Our patient had two important risk factors: age at onset and positivity for anti-TIF1γ antibodies.

**Figure 5:**
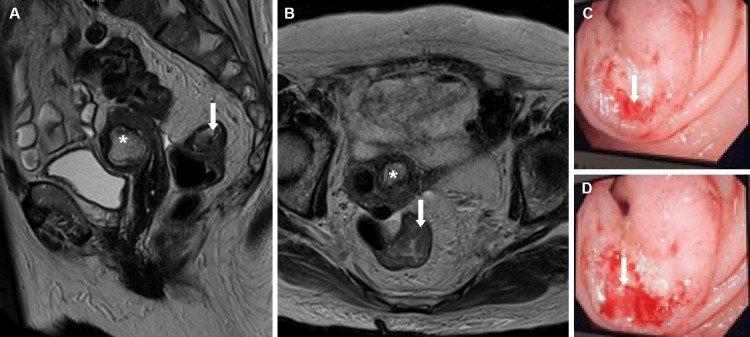
Colorectal mass findings. A and B: T2 MRI scan (sagittal and transverse, respectively) findings of the colorectal lesion. C and D: Colonoscopy findings live in the exam room showing a stenotic, friable lesion in the colorectal transition. Bold arrow: neoplastic colorectal lesion. Asterisk: calcified leiomyoma of the uterus.

Thoracic-abdominal-pelvic CT scan showed colorectal transition thickening at 47 mm, 16 cm above the anal margin, with no adenopathies or secondary deposits (Figure 5A, 5B). No findings were suggestive of interstitial lung disease, which sometimes presents in DM and Sjögren’s syndrome. Colonoscopy identified a vegetative lesion occupying 75% of the circumference of the lumen, friable and with loss of crypt and vascular pattern, suspicious for atypia 16 cm above the anal margin (Figure 5C, 5D).

**Figure 6:**
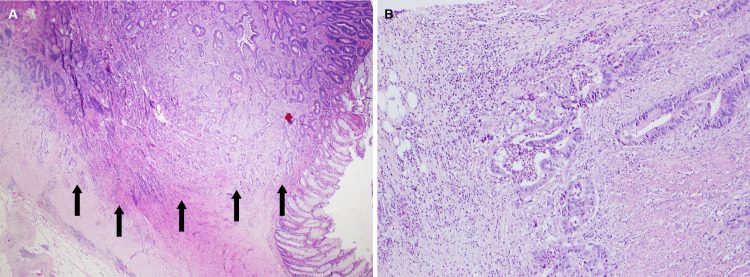
Colorectal adenocarcinoma with adjacent normal mucosa (hematoxylin-eosin staining) A: small magnification, B: high magnification. Black arrows demonstrate colorectal adenocarcinoma.

Biopsies were taken, confirming the diagnosis of adenocarcinoma, moderately differentiated (G2), MSI-stable (MSS) (Figure 6A, 6B).

To complete staging, a pelvic MRI was done, showing the lower invasion point to be 14 cm above the anal margin - thus invading the rectum - with possible contact with anterior rectum peritoneal reflection. No secondary deposits or adenopathies were found. Cancer was staged as IIIB rectal adenocarcinoma (T4N0M0). Carcinoembryonic antigen (CEA) was 3.9 ng/mL.

Treatment

DM and Sjögren’s Syndrome Medical Treatment

**Figure 7:**
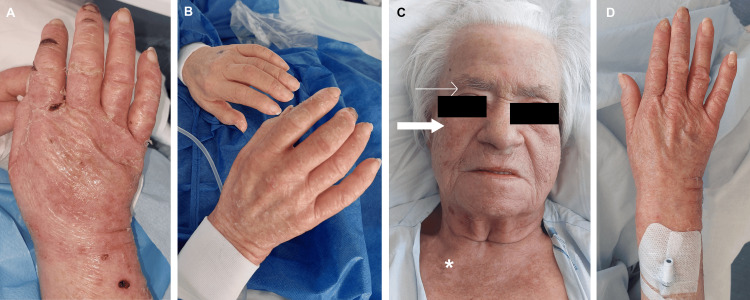
Improvements of hand and facial cutaneous lesions throughout the treatment. A: After three days of therapy with topical vaseline, betamethasone, and oral acetylsalicylic acid, with marked skin peeling. B: After 13 days of the same therapy, already with no fissures or skin peeling. C: Facial lesions after surgery showing no edema, heliotrope rash (narrow arrow), or malar rash (bold arrow), normalization of capillary implantation line, but with the persistence of poikiloderma (asterisk). D: Hand in the same time point as C showing no edema, erythema, or ulcers.

While investigations were carried out, acetylsalicylic acid 100 mg id and topical betamethasone three times a week and vaseline twice daily were applied to the face and hands, as recommended by the Dermatology department (Figure 7A). After 13 days, there was already some improvement mainly in the hands (Figure 7B).

After myositis-specific antibodies were positive and the biopsy was performed, the patient was started on prednisolone id (7.5 mg) and hydroxychloroquine id (200 mg alternated with 400 mg every other day), with good tolerance and significant improvement. Side effects included only mild diarrhea.

Cancer-Directed Surgery

The multidisciplinary board decided on a direct cancer resection. As glucocorticoid therapy halts the post-surgery cicatricial process, the patient was submitted to gradual weaning of corticoids and hydroxychloroquine until ready for surgery. Interruption of therapy led to the recurrence of cutaneous lesions. Meanwhile, prehabilitation physiotherapy was performed in order to reduce post-surgery complications and improve prognosis [13].

A Hartman’s procedure was performed, without major complications, except superficial surgical wound dehiscence, which was promptly corrected. Pathological analysis revealed a moderately differentiated rectal adenocarcinoma, which invaded the muscularis propria into the pericolonic tissue. It was completely resected (R0). One regional lymph node metastasis, out of the 25 resected regional lymph nodes, was documented. The final pathological staging, using AJCC 8th edition, was pT3N1aM0 - IIIB. The multidisciplinary board opted for a watch-and-wait strategy, with general surgery follow-up appointments.

After surgery, medical therapy with hydroxychloroquine was immediately restarted, and corticotherapy was reinstated after 35 days. Vaseline twice daily and betamethasone three times a week were maintained. Improvements in cutaneous lesions (Figure 7C, 7D), pruritis, and quality of life were notorious.

Follow-up

The patient retook physiotherapy in the post-op as soon as clinically possible before discharge, with good tolerance.

After hospital discharge, the patient went to a rehabilitation clinic, taking physiotherapy and occupational therapy, to regain her ability to walk alone and overall autonomy. The patient's previous capabilities were mostly recovered.

At six months post-op appointment, there were no clinical complaints, CEA was 2.2 ng/mL, and no local or distant recurrence of neoplastic disease was found on the thoracic-abdominal-pelvic CT. No evidence of DM relapse was found. She was able to walk by herself, with the help of a walking stick.

## Discussion

DM must be identified as soon as possible considering its frequent association with malignancy and evolution with complications as an interstitial lung disease and dysphagia. However, establishing the diagnosis is still a clinical challenge, with frequent delays. This patient had started symptoms 13 months before and had gone to several general practitioner appointments before coming to our emergency department. Similar delays are described in the literature, with a median of 15.5 months until diagnosis [14]. Early diagnosis might have avoided rectum cancer progression (already a stage IIIB, with lymphatic invasion) and might have improved the patient’s quality of life. In other cases, repercussions can be even starker.

The most well-known presentation of dermatomyositis includes cutaneous typical lesions and symmetric muscle weakness (classic dermatomyositis). The patient we described had a collection of both cutaneous findings and proximal muscle weakness, although no elevations of muscle enzymes or electromyogram myositis changes were detected. However, a few cases of classic DM without muscle enzyme elevation or without electromyogram changes have been reported [15,16]. In a study of 72 patients with dermatomyositis, creatine kinase (CK), lactate dehydrogenase (LDH), aspartate transaminase (AST), and alanine transaminase (ALT) were reported to be within a normal range in just 1.3% of patients, whereas electromyogram was completely normal in 10.7% patients, with an otherwise classical disease [15]. We hypothesize that our patient either presented with a rare form of classic DM without elevation of muscle enzymes or that her muscle weakness stems from the Sjögren syndrome. Given that her muscle weakness improvement was gradual and involved intensive physiotherapy pre- and post-tumor resection, causality regarding its origin is difficult to establish.

Regarding muscle biopsy changes in DM, these are varied but include perimysial and/or perivascular inflammation, perifascicular atrophy, degenerating and regenerating fibers, and perifascicular elevation of MHC class 1. In this case, findings comprised myophagocytosis, necrosis, and variation in the muscle fiber size, with global MHC-I staining. These represent nonspecific inflammatory myopathy and can be found both in DM and Sjögren-associated myositis. Unfortunately, there was not sufficient sample material to further study the muscle.

DM is associated with several myositis-specific antibodies. These are usually associated with characteristic clinical features. Anti-TIF1γ antibodies are strongly associated with an increased risk of cancer (9.37-fold risk, especially solid cancer with advanced staging) and exacerbated skin symptoms [8-10]. Indeed, the patient we described had a locally advanced rectal cancer and exuberant skin lesions.

The occurrence of Sjögren’s syndrome is more likely in patients who have already a systemic rheumatologic disease, including DM, compared to a healthy population. The literature estimates an incidence of Sjögren’s syndrome 5.9-fold higher than the general population [17]. Our patient complained of local symptoms, including xerophthalmia, xerostomia, frequent oral aphthous ulcers, a burning mouth sensation, depapilated dry tongue and cheilitis (Figure 1A), and systemic symptoms, including fatigue.

The British Society for Rheumatology guidelines for idiopathic inflammatory myopathy state that malignancy screening must be done provided the patient has one of the following risk factors referred before. Evidence suggests the use of chest/abdomen/pelvis CT scans and in selected cases PET/CT scans and tumor markers [11]. Our patient underwent malignancy screening with a thoracic-abdominal-pelvic CT scan, which found a mass, and further workup was done accordingly.

Paraneoplastic DM management is not straightforward. No guidelines define the best timing for surgery or medical treatment [13]. Glucocorticoids are the cornerstone of the initial medical treatment to avoid muscle complications and improve strength. Other corticoid-sparing agents can then be started after the initial treatment or, according to some authors, can be started right away. Hydroxychloroquine is an antimalarial effective in controlling skin diseases in up to 75% of cases. In cancer-associated DM, it is important to treat the cancer to appropriately manage the disease [12]. Our patient improved with both these agents, corticosteroids and hydroxychloroquine, with cutaneous symptoms having resolved after surgery and restart of the treatment.

DM management in the geriatric population (≥65 years old) must take into consideration several factors. Dysphagia is more frequently reported, and cancer diagnoses - especially carcinomas - are more commonly found, as in our patient [18]. No differences between muscle or cutaneous manifestations are described [18]. Erythrocyte sedimentation rate, C-reactive protein, ferritin, and fibrinogen are often higher in the elderly population with this condition [18]. Prognosis is poorer [18,19,20]: fewer older patients achieve remission (~14%) compared to younger counterparts (~41%), and mortality rates are significantly higher (48% vs. 7%) [18]. Therefore, in this subset of patients, intensification of therapy and close follow-up are recommended [18]. Our very elderly patient, however, seems to have achieved remission with a mild corticotherapy dose, hydroxychloroquine, and surgery. We hypothesize that adjuvant hydroxychloroquine and, above all, cancer resection have played a crucial role, as suggested chronologically by the great improvement after it.

## Conclusions

Anti-TIF1γ-DM is a cancer red flag. Prompt recognition of DM clinical, analytic, and electrophysiologic changes contributes to better patient outcomes, including earlier detection of associated neoplasms and faster resolution of dermatomyositis. However, as highlighted in this case report, DM must not be discarded upon normal enzymes and electromyogram findings. Indeed, clinically amyopathic dermatomyositis is a possible diagnosis, and further studies should eventually be carried out to evaluate whether classic dermatomyositis with normal muscular enzymes and electromyogram can occur.

Medical treatment of cancer-associated dermatomyositis is not straightforward. It includes DM medical treatment with a combination of corticosteroids, hydroxychloroquine, or corticosteroid-sparing agents, as well as cancer-directed treatment and control of concomitant rheumatologic diseases. Older patients have poorer prognosis and may require intensification of therapy and closer follow-up. Resolution of DM often provides great relief to patients since it can be severely incapacitant due to pruritus, cutaneous lesions, and musculoskeletal limitations.
